# Assessing the potential relevance of CEACAM6 as a blood transcriptional biomarker

**DOI:** 10.12688/f1000research.126721.1

**Published:** 2022-11-11

**Authors:** Darawan Rinchai, Damien Chaussabel

**Affiliations:** 1 St. Giles Laboratory of Human Genetics of Infectious Diseases, The Rockefeller University, New York, New York, 10065, USA; 2 Computer Sciences Department, The Jackson Laboratory, Farmington, CT, 06032, USA

**Keywords:** Biomarkers, CEACAM6, Transcriptional profiling, Literature profiling

## Abstract

Background

Changes in blood transcript abundance levels have been associated with pathogenesis in a wide range of diseases. While next generation sequencing technology can measure transcript abundance on a genome-wide scale, downstream clinical applications often require small sets of genes to be selected for inclusion in targeted panels. Here we set out to gather information from the literature and transcriptome datasets that would help researchers determine whether to include the gene CEACAM6 in such panels.

Methods

We employed a workflow to systematically retrieve, structure, and aggregate information derived from both the literature and public transcriptome datasets. It consisted of profiling the CEACAM6 literature to identify major diseases associated with this candidate gene and establish its relevance as a biomarker. Accessing blood transcriptome datasets identified additional instances where CEACAM6 transcript levels differ in cases vs controls. Finally, the information retrieved throughout this process was captured in a structured format and aggregated in interactive circle packing plots.

Results

Although it is not routinely used clinically, the relevance of CEACAM6 as a biomarker has already been well established in the cancer field, where it has invariably been found to be associated with poor prognosis. Focusing on the blood transcriptome literature, we found studies reporting elevated levels of CEACAM6 abundance across a wide range of pathologies, especially diseases where inflammation plays a dominant role, such as asthma, psoriasis, or Parkinson’s disease. The screening of public blood transcriptome datasets completed this picture, showing higher abundance levels in patients with infectious diseases caused by viral and bacterial pathogens.

Conclusions

Targeted assays measuring CEACAM6 transcript abundance in blood may be of potential utility for the management of patients with diseases presenting with systemic inflammation and for the management of patients with cancer, where the assay could potentially be run both on blood and tumor tissues.

## Introduction

Changes in blood transcript abundance can reflect differences in relative abundance of leukocyte populations as well as transcriptional regulation secondary to immune activation (for instance inflammation, interferon, and prostaglandin responses). Quantifying these changes can thus be relevant for making clinical decisions. ^1^^,^^2^ Robust technology platforms, such as microarrays and RNA sequencing, that enable the measurement of transcript abundance in an unbiased fashion (i.e., simultaneously measuring all RNA species that are present in a given sample) have been widely available for the past two decades. As a result, blood transcriptome studies have been conducted across a wide range of pathological or physiological states. ^3^^–^^7^ In addition, vast amounts of blood transcriptome profiling data have been made available in public repositories such as the NCBI Gene Expression Omnibus, or EMBL-EBI’s array express. ^8^

Transcriptome profiling data can be leveraged to inform the design of targeted gene panels. These panels can serve as a basis for the development of diagnostic assays for use in clinical settings. But targeted assays can also be employed in research settings, for instance when profiling of transcript abundance needs to be performed on large scales (e.g., in thousands of samples) and with a relatively short turnaround. Notably, targeted assays could also prove valuable in resource-constrained settings, where computing infrastructure, instrument, and reagents costs are limiting. The approaches employed for targeted assay design can be data-driven (e.g., applying computational models to transcriptome profiling dataset(s) to select genes based on their predictive performance) or knowledge-driven (selecting genes based on pre-existing knowledge – e.g., for the development of an “immunology panel”). However, both data and knowledge-driven approaches can also be combined. This is illustrated in recently published work in which we describe the selection of three blood transcriptional panels designed for the monitoring of responses to SARS-CoV-2. ^9^ Transcripts were selected first based on their membership to co-expressed gene sets, the abundance of which was found to change during COVID-19 disease (i.e., through a data-driven approach) and second based on their relevance to one of three themes, which were immunity, therapeutic development, and severe acute respiratory syndrome biology (i.e., through a knowledge-driven approach). However, the amount of information available in the literature and in public transcriptome datasets that can be leveraged for candidate gene selection can be overwhelming. Thus, we have developed an approach to identify, retrieve, structure, and aggregate such information in a manner that would support the rational selection of candidate genes for inclusion in targeted assays destined to be used in clinical or research settings. ^10^

Here we decided to focus on CEACAM6, a gene encoding a protein of the carcinoembryonic antigen (CEA) family whose members are glycosylphosphatidylinositol (GPI)-linked cell surface proteins. ^11^^,^^12^ We employed an approach devised as part of a “collective omics data” (COD) training curriculum, ^13^ relying in particular on the recently published COD1 training module workflow. ^10^ Briefly, it consists of: 1) selecting a gene and retrieving background information and associated literature; 2) profiling its literature at a high level, reporting instances where it has been associated with diseases, and its relevance as a biomarker in such settings; 3) profiling the literature in more depth and reporting changes in abundance for transcripts encoded by this gene measured in whole blood; 4) profiling the abundance of the candidate gene across multiple relevant transcriptome datasets; 5) preparing a manuscript and submit it for peer-review and publication. Throughout these steps trainees parse the information being retrieved (e.g., from the title or text of articles) and record it in a structured format (i.e., a spreadsheet). They also aggregate this information in interactive circle packing plots, which are collections of circular elements used to visualize a hierarchic organization, with access to each level being achieved through zoom-in and -out functionalities.

Screening the CEACAM6 literature identified a strong association with various cancers, in particular colorectal cancer where measurement of CEACAM6 blood transcript levels may be of clinical value for early detection. ^14^^,^^15^ Associations were also found for pancreatic, lung, and breast cancer, as well as leukemia and inflammatory bowel disease. More in depth profiling of the literature (analyzing the full text) identified an array of conditions for which CEACAM6 abundance has been found to be significantly different from controls. This list was complemented by a screening of public blood transcriptome datasets. The tables employed to capture this information in a structured format are shared as extended data files. Another deliverable is the interactive circle packing plot that permits aggregation and seamless access to this and all underlying information. Altogether these resources supported manuscript preparation and interpretation/evaluation by the authors of the relevance of CEACAM6 as a biomarker. They may also support transcript selection efforts of members of the research community interested in designing blood transcriptional biomarker panels.

## Methods

### Overall literature and large-scale dataset profiling approach

The workflow implemented here to assess the potential of CEACAM6 as a blood transcriptional biomarker has been described in detail in a separate methods paper. ^10^ The approach was devised as part of a training module focused on the development of skills for the retrieval, structuring, aggregation, and interpretation of information derived from the literature and publicly available large-scale profiling datasets. Relevant resources that have been employed and generated in the context of this work are presented in Table 1.

**Table 1.** List of online resources employed for profiling CEACAM6 literature/transcriptional data, including those generated as part of the present work.

Resource name/Description	Use	Link	Reference
CEACAM6 Interactive Circle Packing Plot	Aggregation and dissemination of information derived from the literature and transcriptional data profiling efforts	https://prezi.com/view/pQ7TKEC6tgY3cuik9ckt/	Present work
Generic information capture form	Excel spreadsheet employed for structuring relevant information captured via the screening of CEACAM6 literature or transcript abundance profiles	https://doi.org/10.6084/m9.figshare.21183718.v1	Present work, Extended Data File 1 ^19^
Prevalence of disease or cell type entities in the CEACAM6 literature	Prioritization of disease or cell types associated with CEACAM6	https://doi.org/10.6084/m9.figshare.21183748.v1	Present work, Extended Data File 2 ^29^
Information captured from the literature identifying CEACAM6 as a candidate biomarker	This information can be used as a basis for deciding whether to include CEACAM6 in a targeted panel	https://doi.org/10.6084/m9.figshare.21183832.v1	Present work, Extended Data File 3 ^30^
Information captured from the literature identifying instances where abundance levels of CEACAM6 blood transcripts differ between cases and controls	This information can be used as a basis for deciding whether to include CEACAM6 in a targeted panel	https://doi.org/10.6084/m9.figshare.21184357.v1	Present work, Extended Data File 4 ^55^
Transcript abundance measurements for CEACAM6 across 16 reference transcriptome datasets	Determining CEACAM6 differential expression and generating graphical representations	https://doi.org/10.6084/m9.figshare.21184363.v1	Present work, Extended Data File 5 ^56^
Information captured from reference blood transcriptional datasets for which CEACAM6 transcripts were found to differ between cases and controls	This information can be used as a basis for deciding whether to include CEACAM6 in a targeted panel	https://doi.org/10.6084/m9.figshare.21184369.v1	Present work, Extended Data File 6 ^57^
Gene Expression Browser (GXB) CD2K instance	Access CEACAM6 abundance profiles across multiple reference datasets	http://cd2k.gxbsidra.org/dm3/geneBrowser/list	^20^
Single Cell Portal	Identification of scRNAseq datasets where CEACAM6 expression is elevated in one or several cell clusters	https://singlecell.broadinstitute.org/single_cell	^61^

Briefly, the process is broken down into the following steps:

(1) Selecting a candidate gene: the most basic criterion is for transcripts for this gene to be detectable in blood. It could also be selected based on its membership in a pre-defined signature or gene set.
(2) Retrieving background information: background information about the gene is gathered from reference datasets (e.g., OMIM [ https://www.omim.org/], UniProt [ https://www.uniprot.org/], Entrez Gene [ https://www.ncbi.nlm.nih.gov/gene]) and the introduction section of recent publications.
(3) Profiling the candidate gene’s literature at a high level: the literature associated with the candidate gene is identified (see “literature profiling section” below for details). Entities corresponding to a given theme (e.g., diseases, cell types, or molecular processes) are extracted from the title of those articles (“breast cancer” is an example of a disease entity). This permits to identify the main diseases associated with the gene of interest, and, in turn, identify instances in which the candidate gene has been found to be of actual or potential utility as a biomarker for these diseases.
(4) Profiling the literature in more depth: taking advantage of Google Scholar’s full text search capabilities, this step identifies publications where the abundance level of the candidate gene’s transcripts in blood samples was found to be different in patients compared with appropriate controls.
(5) Profiling the abundance of the gene across multiple relevant transcriptome datasets: to complement the previous step, public blood transcriptome datasets are screened to identify instances where the abundance level of the candidate gene’s transcripts in blood differs in patients in comparison with appropriate controls.
(6) Developing resources supporting manuscript preparation and evaluation of the candidate gene: the information parsed from the literature or transcriptome datasets in earlier steps is recorded in a structured format (e.g., using a standard spreadsheet template, see details below). Using the Prezi web application (Prezi Inc., San Francisco, CA, USA), this information is aggregated in interactive circle packing plots. Spreadsheets and interactive circle plots can next be used to assess the overall relevance of the gene of interest as a candidate blood transcriptional biomarker and support the writing of the manuscript. They can also serve as a resource for investigators interested in designing blood transcriptional biomarker panels.

### BloodGen3 blood transcriptional module repertoire

CEACAM6 was selected based on its membership to one of the 382 modules constituting the fixed BloodGen3 module repertoire. This repertoire has been recently characterized. ^16^ Briefly, it was constructed based on co-expression analysis through a process that was exclusively data-driven. First, the 16 reference blood transcriptome datasets that served as input were clustered separately using K-means clustering. Co-clustering events observed across the 16 reference datasets were then recorded for each gene pair. This information served as a basis for the constitution of a large co-clustering network, with nodes representing genes and edges representing co-clustering events. A weight of 1 to 16 was attributed to the graph edges depending on the number of times co-clustering events were observed. The network was then mined using graph theory to identify densely connected subnetworks that were identified as modules and added to the repertoire. This process eventually yielded 382 non-overlapping modules (at the probe level, multiple probes mapping to the same gene could be found across different modules). Next, the repertoire was thoroughly characterized functionally and an R package was developed to support BloodGen3 module repertoire analysis and visualization. ^17^

### Literature profiling

The approach has been described in two published study guides: from a high-level perspective as part of the COD1 workflow ^10^ and in more detail in a separate study guide dedicated to literature profiling. ^18^ An overview of the steps implemented in the profiling of the literature associated with CEACAM6 is provided here:

(1) Literature retrieval: to identify the literature associated with the candidate gene, a PubMed query is designed by combining the official gene name and symbol along with known aliases. Troubleshooting is performed as needed to minimize false positives and false negatives. For CEACAM6 the following query was generated and, as of August 16 2022, returned 642 entries:

*CEACAM6 [tiab] ORc “CEA Cell Adhesion Molecule 6” [tiab] OR CD66c [tiab] OR (NCA [tiab] AND (Carcinoembryonic OR CEACAM6 OR CD66c)) OR “Carcinoembryonic Antigen-Related Cell Adhesion Molecule 6” [tiab] OR “Carcinoembryonic Antigen Related Cell Adhesion Molecule 6” [tiab] OR “Carcinoembryonic Antigen-Related Cell Adhesion Molecule 6” [tiab] OR (“Normal Cross-Reacting Antigen” [tiab] AND (Carcinoembryonic OR CEACAM6 OR CD66c)) OR (“Non-Specific Cross-reacting Antigen” [tiab] AND (Carcinoembryonic OR CEACAM6 OR CD66c)) OR (CEAL [tiab] AND (Carcinoembryonic OR CEACAM6 OR CD66c)) NOT review [pt]*

(2) Extraction of relevant concepts: the titles of the articles associated with CEACAM6 are screened for keywords associated with diseases or physiological states and with cell types. For example, if the theme is “diseases or physiological states”, diseases entities such as “breast cancer”, “influenza infection”, “pregnancy” or “systemic lupus erythematosus” may be identified in the title of articles associated with the gene of interest.
(3) Generating literature profiles: next, the prevalence of the cell types or disease entities identified in the previous step in the candidate gene’s literature is determined. Focusing on a subset of the literature, information regarding the potential relevance of the candidate gene as a biomarker can be captured in a structured format in an Excel spreadsheet.
(4) Aggregating information: the underlying literature profiling information is captured and visually represented in interactive circle packing plots using the Prezi application (Prezi Inc, San Francisco, CA, USA). This serves as a basis for generating manuscript figures and the constitution of a companion resource that can be made accessible to the community.

### Information retrieval and structuring

While screening the literature and large-scale profiling datasets trainees learn to identify and extract key information from research articles or transcriptome datasets. These include basic information, as well as elements of study design (e.g., analyte name, type, species, biological samples, measurement methods, sample size) and findings (e.g., fold change, significance). The information is captured in a standard MS Excel spreadsheet template, which can be used to record information derived from both the literature and transcriptome profiling datasets ( **Extended Data File 1** ^19^).

### Interactive circle packing plots

Information extracted from the literature and from public transcriptome datasets was aggregated in an interactive circle packing plot generated using the Prezi web application (Prezi Inc., San Francisco, CA, USA). A free basic Prezi account can be setup for this ( https://prezi.com/pricing/basic/). Starting from a blank presentation, it consisted of adding and populating circles (topics) and organizing them into a hierarchy ( https://prezi.com/view/pQ7TKEC6tgY3cuik9ckt/). Color-coding the circles and varying their size permitted the visualization of some of the results. Excerpts or full articles were added, as well as plots representing CEACAM6 transcriptional data profiles. Links to articles and interactive versions of the figures were also provided in order promote seamless access to information.

### Transcriptome profiling data analyses and visualization

Screening of transcriptome profiling datasets consisted of determining whether differences between levels of CEACAM6 transcript abundance in patients and their respective controls were significant. The CEACAM6 profiling data were downloaded from the “CD2K” gene expression browser (GXB) instance ( http://cd2k.gxbsidra.org/dm3/geneBrowser/list) for multiple blood transcriptome datasets. ^20^ Analyses were conducted separately for each dataset in Microsoft Excel (RRID:SCR_016137), testing for differences in variance using F-test statistics and testing for differences in expression using t-test statistics. Differences were considered significant when p was <0.05. Plots were generated using Plotly chart studio (RRID:SCR_013991, https://chart-studio.plotly.com/create/).

## Results

### Selection of CEACAM6

**Figure 1.**
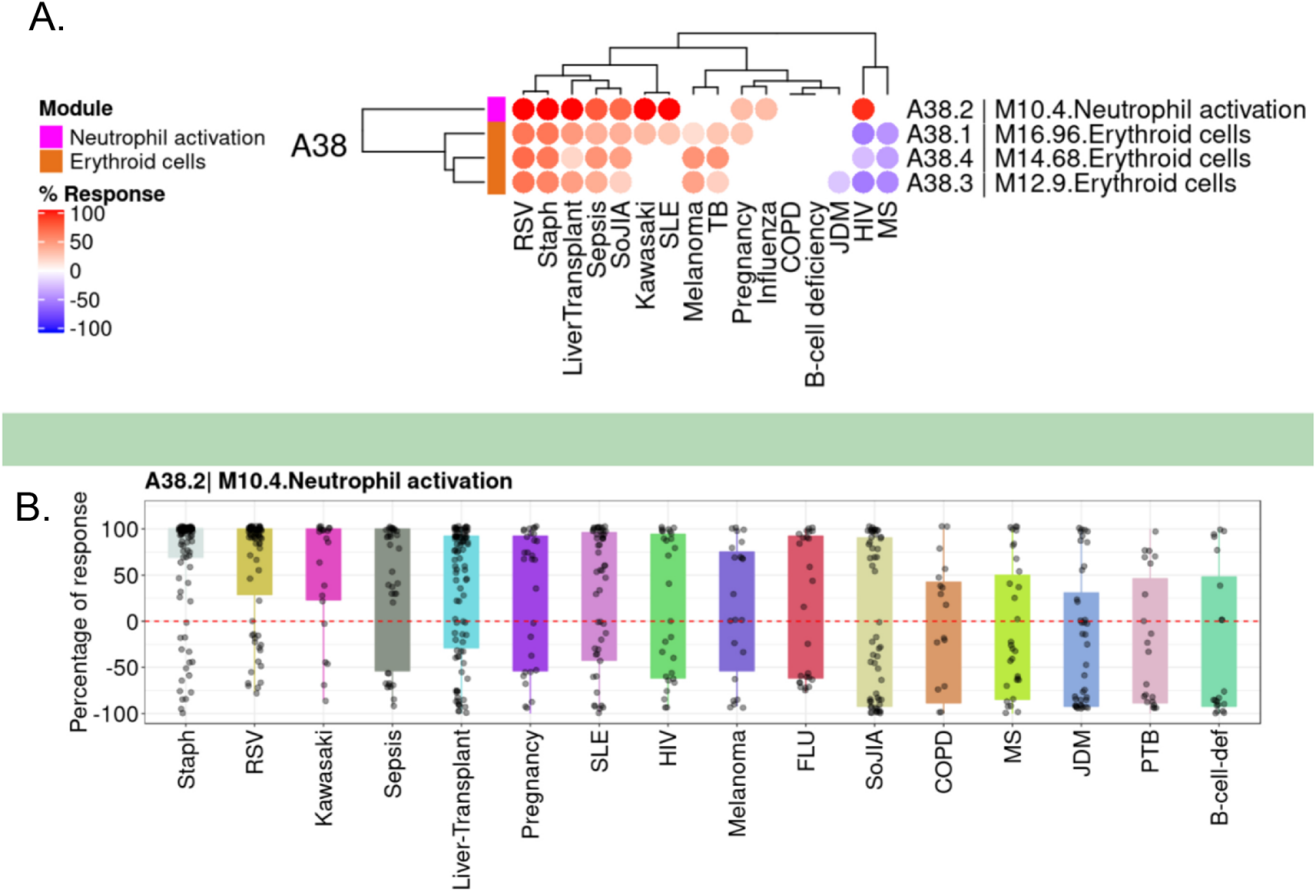
Differences in transcript abundance levels for BloodGen3 module M10.4 across 16 reference datasets. A. The module fingerprint heatmap represents the proportion of transcript for a given module (rows) for which abundance levels are significantly different in case subjects compared to the respective controls for a given reference dataset (columns). Values can range from +100% (solid red: abundance for all constitutive transcripts for the module are significantly higher) to −100% (solid blue: abundance for all constitutive transcripts for the module are significantly lower). Responses are shown for four modules included in the module aggregate A38 from the BloodGen3 repertoire, ^16^ including module M10.4 from which CEACAM6 was selected. B. The box plot represents the percentage response averaged for module M10.4, across the 16 reference datasets (we have contributed this dataset collection to GEO as part of an earlier work, ^16^ and it is accessible under accession number GSE100150. Plots were generated using the BloodGen3 web application: https://drinchai.shinyapps.io/BloodGen3Module/.

The first step consisted of selecting a gene that would be next evaluated for its potential relevance as a blood transcriptional biomarker. CEACAM6 was selected primarily based on its membership to a blood transcriptional signature of interest. This signature is part of a fixed blood transcriptional module repertoire (BloodGen3, see Ref. 16 and methods for details). The M10.4 module signature is functionally associated with neutrophil activation and comprises 11 other genes: BPI, LTF, CEACAM8, DEFA1, DEFA1B, DEFA2, DEFA4, OLFM4, ELANE, CTSG, and MPO ( https://prezi.com/view/pQ7TKEC6tgY3cuik9ckt/ Step 1: candidate gene selection). In a reference collection of 16 patient cohorts, ^16^ abundance levels of M10.4 transcripts were the highest in subjects with *Staphylococcus aureus* infection, respiratory syncytial virus infection and bacterial sepsis ( Figure 1).

### General background information about CEACAM6

As part of the evaluation process, it can be useful to start by retrieving and synthesizing background information about the candidate gene. For this, summaries from different reference databases, as well as introductions from recent publications on CEACAM6, were retrieved. This information was recorded in the CEACAM6 interactive circle packing plot ( https://prezi.com/view/pQ7TKEC6tgY3cuik9ckt/ Step 2: gathering background information) and used for development of the narrative below.

CEACAM6 is a glycosyl phosphatidyl inositol (GPI)-anchored cell surface glycoprotein. It is a member of the carcinoembryonic antigen (CEA) family whose members are known to play a role in cell adhesion. ^21^ Specifically, CEACAM6 expression has been reported in granulocytes and lung and intestinal epithelial cells. ^22^ In ileal epithelial cells of patients with Crohn’s disease, CEACAM6 has been found to act as a receptor for adherent-invasive *Escherichia coli.* ^23^ It has also been found to mediate entry of *Neisseria gonorrhoeae.* ^24^ CEA family members are widely used as tumor markers in serum as well as tumor immunoassays. CEACAM6 has been reported to act as an oncogene, promoting tumor progression and metastasis. ^25^ These properties may, at least in part, be effected via the role of CEACAM6 in promoting anoikis resistance, which prevents the homeostatic elimination of anchorage-dependent cells (such as epithelial cells) that are detached from the cellular matrix. ^26^ Since CEACAM6 membrane expression is highly specific to tumor cells, it has been suggested as a target for different cancer immunotherapies. ^27^ It has also recently been identified as an immune checkpoint molecule, based on its role in suppressing cytotoxic T cell responses against malignant plasma cells. ^28^

### Profiling the CEACAM6 literature at a high-level reveals an association with neutrophils and several types of cancers

To further our understanding of the biological significance and clinical relevance of CEACAM6, we next sought to systematically screen the literature to identify associations with cell populations and diseases or physiological states.

**Figure 2.**
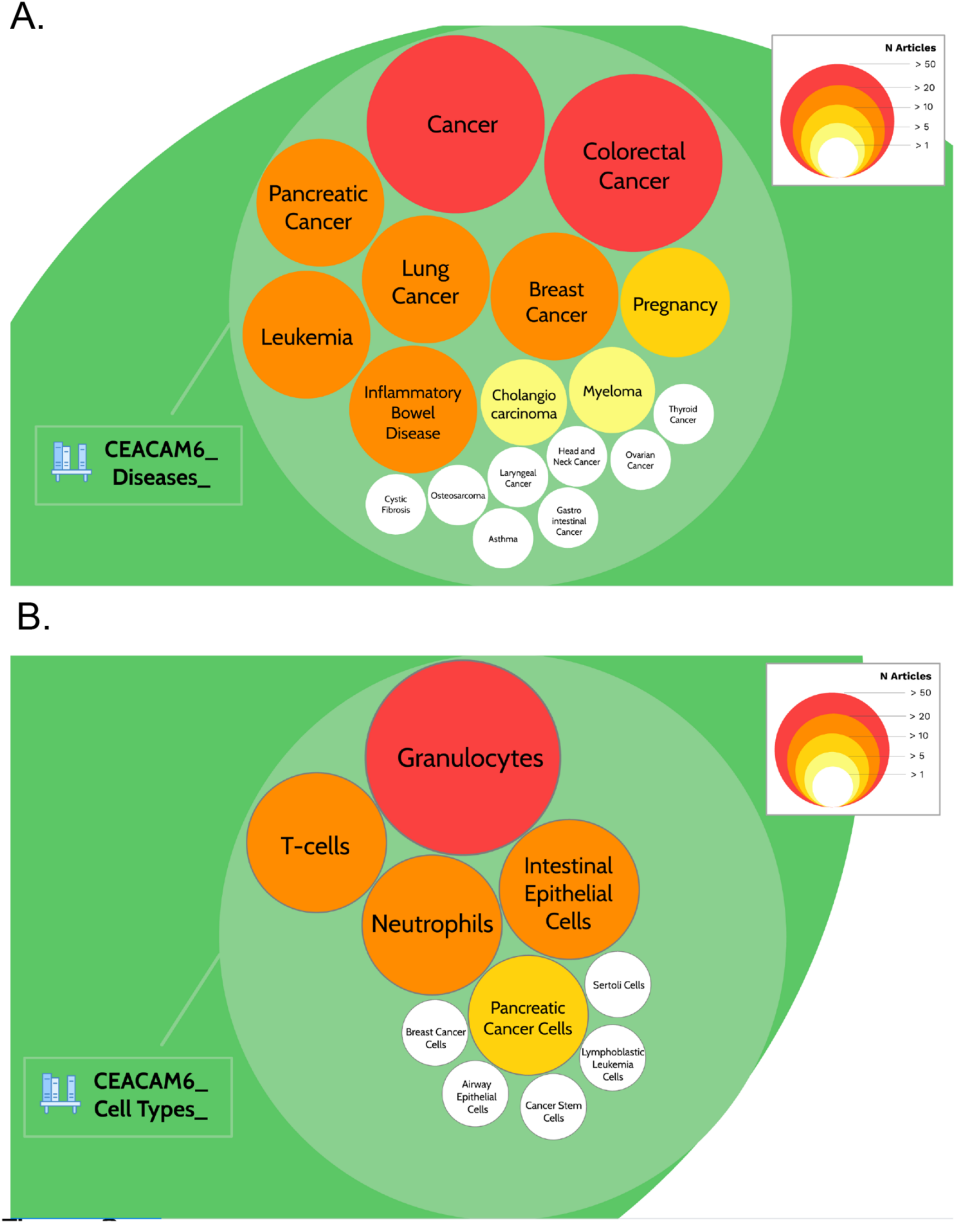
CEACAM6 disease and cell type literature profiles. The prevalence of articles among the literature associated with CEACAM6 for disease entities (A) or cell type entities (B) are represented by circles of different sizes and colors, corresponding to the number of associated articles. It is possible to access underlying information by zooming into each of the circles. The Prezi presentation can be accessed at this url: https://prezi.com/view/pQ7TKEC6tgY3cuik9ckt/ Step 3: background literature profiling.

A query was designed to permit the retrieval of the literature associated with CEACAM6 (see methods for details). In total 642 PubMed entries were returned. Screening for names of diseases in the titles of literature associated with CEACAM6 identified 18 entities ( **Extended Data File 2** ^29^). Among these, “cancer” and “colorectal cancer” were found in more than 50 CEACAM6-associated articles (202 and 65, respectively, as of March 2022). “Pancreatic cancer”, “lung cancer”, “breast cancer”, leukemia” and “inflammatory bowel disease” were found in more than 20 CEACAM6-associated articles (31, 35, 28, 35, and 30, respectively; Table 2, Figure 2A & https://prezi.com/view/pQ7TKEC6tgY3cuik9ckt/: Step 3/CEACAM6_Diseases). “Pregnancy” was found in 14 CEACAM6-associated articles. “Cholangiocarcinoma” and “myeloma” were found in more than 5 articles (7 and 9, respectively). Eight other diseases were found in only one article. Screening titles for names of cell types identified 10 entities ( **Extended Data File 2** ^29^). The most frequently mentioned cell types among the CEACAM6 literature were granulocytes, neutrophils, T-cells and intestinal epithelial cells ( Table 2, Figure 2B & https://prezi.com/view/pQ7TKEC6tgY3cuik9ckt/: Step 3/CEACAM6_Cell Types).

**Table 2.** List of the most prevalent diseases/physiological states and cell types found among the CEACAM6 literature.

Themes	Entities	N articles	% CEACAM6 Literature
Diseases/physiological states	Colorectal cancer	65	10.1%
Diseases/physiological states	Pancreatic cancer	31	4.8%
Diseases/physiological states	Lung cancer	35	5.5%
Diseases/physiological states	Leukemia	35	5.5%
Diseases/physiological states	Inflammatory bowel disease	30	4.7%
Diseases/physiological states	Breast cancer	28	4.4%
Cell types	Granulocytes	66	10.3%
Cell types	Neutrophils	43	6.7%
Cell types	T-cells	27	4.2%
Cell types	Intestinal epithelial cells	26	4%

Altogether, this step established that CEACAM6 is associated with a large body of literature. It also permitted the identification of the main cell types and diseases associated with this gene. This information was used in subsequent literature profiling steps.

### CEACAM6 is of potential clinical relevance in the diagnosis of cancers, in particular, the early detection of colorectal carcinoma

The selection of a blood transcriptional panel could take into consideration whether a given candidate gene has already been determined to be of clinical relevance as a biomarker, whether that is at the gene, transcript, or protein level. Thus, we next sought to determine if this was the case for CEACAM6 by extracting relevant information from its literature for the main disease entities identified in the previous steps.

The approach is described in detail in the methods section. In brief, starting from the CEACAM6-associated literature we searched for publications reporting the actual or potential use of CEACAM6 as a biomarker. For this we focused more specifically on the diseases that showed the highest degree of association with CEACAM6 based on the above literature profiling results (i.e., diseases mentioned in more than 20 articles, which are listed in Table 2), namely: leukemia, colorectal, pancreatic, lung, and breast cancers, as well as Inflammatory bowel disease. Next, articles associated with CEACAM6 and these diseases that also mentioned “biomarker”, “diagnostic”, “diagnosis”, “prognostic” OR “prognosis” in their title or abstract were retrieved. For articles deemed to be of interest, a standard spreadsheet template was used to capture relevant information ( **Extended Data File 3** ^30^). Information was also aggregated in an interactive circle packing plot using the Prezi web application ( https://prezi.com/view/pQ7TKEC6tgY3cuik9ckt/ CEACAM6/Step3: background literature profiling/CEACAM6_Diseases_Biomarker). Together, the information thus gathered served as a basis for the development of the narrative below.

As aforementioned, CEACAM6 has been noted for its oncogenic properties. Our screening of the CEACAM6 literature, which relates more specifically to its potential relevance as a biomarker in various disease settings, supports this notion. Indeed, a higher abundance of CEACAM6, whether at the transcript or protein level, in tumor tissues or serum was always associated with worse survival (in the case of colorectal, ^31^^,^^32^ breast, ^33^^,^^34^ pancreatic, ^35^^–^^39^ and lung cancers ^40^). Other studies have found CEACAM6 to be of potential value for differential diagnosis of malignant vs benign tumors for breast cancer (with CEACAM6 protein levels measured in breast tissues ^41^) and pancreatic cancer (with CEACAM6 protein levels measured in the bile ^42^). Notably, and of particular relevance to this report, in the case of colorectal carcinoma, measuring the abundance of CEACAM6 at the protein and transcript levels in blood alongside TSPAN8, LGALS4, and COL1A2 has been found to be of potential value for early disease detection. ^14^^,^^15^ Furthermore, recently CEACAM6 was also included in a 10-gene signature predictive model for lung cancer prognosis. ^43^

Altogether, this review of the literature shows that measurement of CEACAM6, whether at the transcript or protein level, in tumor tissues or in blood, is considered of potential clinical value in informing the management of different types of cancers, as summarized in Table 3.

**Table 3.** Published reports describing CEACAM6 as being of clinical relevance as a biomarker.

Immune state/pathology	Evidence	Analyte	Sample type	Change	PMID/Ref	Clinical relevance
Colorectal cancer	Literature	mRNA	Blood	Increase	29352642, 26993598 ^14^^,^^15^	Early detection
Colorectal cancer	Literature	mRNA	Tumor	Positive	27042567, 22975528 ^32^^,^^76^	Stem cell marker, Worse prognosis
Colorectal cancer	Literature	Protein	Tumor	Positive	22975528, 14512395 ^31^^,^^32^	Worse prognosis
Pancreatic cancer	Literature	mRNA	Tumor	Positive	34321959 ^35^	Worse prognosis
Pancreatic cancer	Literature	Protein	Serum	Positive	34207784, 25409014 ^36^^,^^37^	Worse prognosis, distant metastases

### In depth screening of the literature shows that blood levels of CEACAM6 transcripts are elevated in a wide range of diseases

More specifically we next sought to assess the relevance of CEACAM6 as a blood transcriptional biomarker. The first pass at screening the literature (above) already identified instances where measuring blood CEACAM6 transcript is deemed of potential clinical value (i.e., for the early detection of colorectal cancer ^14^ or the prognosis of lung cancer ^43^). We wanted to undertake a second pass to profile the literature in more depth to identify additional studies that reported differences in the abundance of CEACAM6 transcripts in blood in patient populations.

Queries were run using Google Scholar, which supports full text search. Entries were screened manually, selecting only peer-reviewed reports where CEACAM6 levels were measured in the blood of human subjects. Relevant information was recorded in a structed format in a spreadsheet using the standard template employed in the previous step. Finally, information was aggregated in the interactive CEACAM6 Prezi circle packing plot.

Differences in CEACAM6 blood transcript levels have been reported in the literature for a wide range of pathologies. Specifically, in addition to the colorectal carcinoma and lung cancer studies described above, it was found to be part of a 13-gene disease signature which was increased in patients with Parkinson’s disease as compared with asymptomatic subject. ^44^ It was also part of a different 13-gene disease signature that was increased in patients with severe idiopathic pulmonary fibrosis compared with patients with a mild form of the disease. ^45^ Notably, other members of this latter signature, including CTSG, DEFA3, and OLFM4, are also comprised in the M10.4 module that is part of the fixed BloodGen3 repertoire mentioned above. Other pathologies and states where blood CEACAM6 transcript levels were found to be increased are summarized in Table 4, and include asthma, ^46^ sepsis, ^47^ post-traumatic stress disorder, ^48^ psoriasis, ^49^ maternal anti-fetal rejection, ^50^ and COVID-19. ^51^^,^^52^ It was also found to differ based on gender (higher in male than in females) ^53^ and notably was also increased by steroid treatment. ^54^ These latter two findings suggest that in instances where demographics or use of steroids are not well-controlled for in the study design, differences in CEACAM6 transcript levels might be, at least in part, attributed to these factors rather than the underlying pathology. For reference, a full record of the information captured from the literature regarding those studies can be found in **Extended Data File 4**. ^55^ Additional information is also found aggregated in the CEACAM6 interactive circle packing plot ( https://prezi.com/view/pQ7TKEC6tgY3cuik9ckt/ CEACAM6/Step3: background literature profiling/CEACAM6_Diseases_Biomarker).

**Table 4.** Pathological, immunological, or physiological states where CEACAM6 transcript abundance levels have been found to differ in cases vs controls.

Disease/physiological state	Evidence	Analyte	Sample type	Abundance levels	PMID/GEO ID
Parkinson’s disease	Literature	mRNA	Blood	Higher	25475535 ^44^
Idiopathic pulmonary fibrosis	Literature	mRNA	Blood	Higher in severe vs mild cases	22761659 ^45^
Psoriasis	Literature	mRNA	Blood	Higher	34639156 ^49^
Colorectal cancer	Literature	mRNA	Blood	Higher	29352642, 26993598 ^14^^,^^15^
Gender difference	Literature	mRNA	Blood	Higher in males	31722210 ^53^
Sepsis non-survivors	Literature	mRNA	Blood	Lower in non-survivors vs survivors	34707398 ^47^
Lung cancer	Literature	mRNA	Blood	Higher levels in patients with poor outcomes	34288383 ^43^
Post-traumatic stress disorder (PTSD)	Literature	mRNA	Blood	Higher levels in PTSD cases associated with increased inflammation vs those without	31698278 ^48^
Maternal anti-fetal rejection	Literature	mRNA	Blood	Lower in fetuses showing evidence of fetal inflammatory response	23905683 ^50^
Steroid treatment	Literature	mRNA	Blood	Higher in patients with Duchenne muscular dystrophy treated with steroids vs those who were untreated	33751844 ^54^
COVID-19	Literature	mRNA	Blood	Higher	35844004 ^52^
COVID-19	Literature	mRNA	Blood	Higher	34335605 ^51^
Asthma	Literature	mRNA	Blood	Higher	27925796 ^46^
Food-induced anaphylaxis	Literature	mRNA	Blood	Higher	26194548 ^77^
Early onset pre-eclampsia	Literature	mRNA	Blood	Lower in patients with early onset pre-eclampsia vs control pregnant subjects	23793063 ^78^
Late onset pre-eclampsia	Literature	mRNA	Blood	Lower in patients with late onset pre-eclampsia vs control pregnant subjects	23793063 ^78^
Female patients with Systemic onset Juvenile Idiopathic Arthritis	Literature	mRNA	Blood	Higher abundance levels in Female SoJIA patients	32794262 ^79^
Kawasaki disease	Public dataset	mRNA	Blood	Higher	GSE100154
Sepsis	Public dataset	mRNA	Blood	Higher	GSE100159
Systemic lupus erythematosus	Public dataset	mRNA	Blood	Higher	GSE100163
*S. aureus* infection	Public dataset	mRNA	Blood	Higher	GSE100165
Pregnancy	Public dataset	mRNA	Blood	Higher	GSE100157
Liver transplant recipients	Public dataset	mRNA	Blood	Higher	GSE100155
Influenza infection	Public dataset	mRNA	Blood	Higher	GSE100160
HIV infection	Public dataset	mRNA	Blood	Higher	GSE100151
RSV infection	Public dataset	mRNA	Blood	Higher	GSE100161

Taken together, this in-depth review of the literature points to differences in CEACAM6 blood transcript abundance being present in patients in a wide range of diseases. Thus, suggests that assays measuring levels of CEACAM6 transcripts in blood may be employed to support biomarker development efforts across different clinical settings.

### Screening of public blood transcriptome datasets to identify elevated levels of CEACAM6 in additional disease settings

Literature reports might capture only a fraction of instances where pathophysiological changes are accompanied by changes in the abundance of CEACAM6 blood transcripts. Screening publicly available transcriptome datasets could confirm published reports and help identify other instances where levels of CEACAM6 transcript abundance differ in patients relative to control subjects.

For this, we employed a data browsing web-application, the Gene eXpression Browser (GXB), ^20^ which provides easy access to transcriptional profiles of individual genes in curated collections of transcriptome datasets. For instance, we screened blood transcriptome data for a collection of 16 reference cohorts that were used for the construction of the BloodGen3 repertoire. These datasets are available in the CD2K instance of GXB ( http://cd2k.gxbsidra.org/dm3/geneBrowser/list). CEACAM6 transcriptional profiles were retrieved for each of these cohorts and statistics run separately using MS Excel to determine the significance of changes in levels of CEACAM6 transcripts in patients vs controls ( **Extended Data File 5** ^56^). Changes were captured in a structured format, plotted, and aggregated in the CEACAM6 circle packing plot.

**Figure 3.**
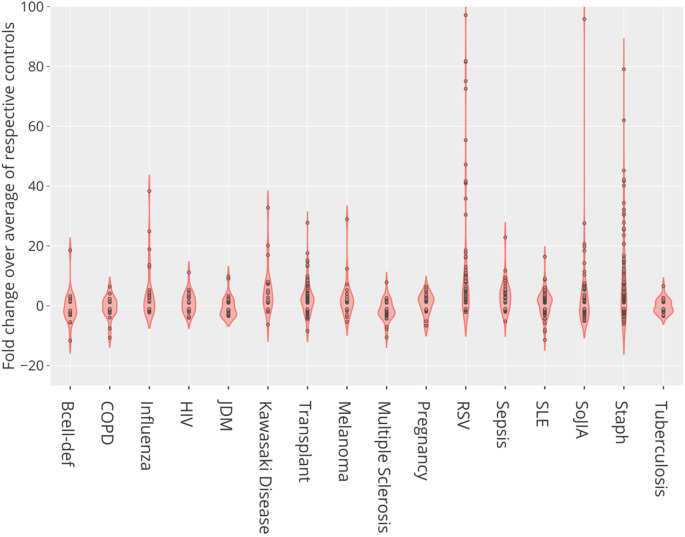
CEACAM6 relative abundance profiles across reference patient cohorts. This violin plot shows the fold change in abundance of CEACAM6 mRNA measured by RNA sequencing in the blood of human subjects across 16 reference disease cohorts, compared to their respective control subjects. In total blood transcriptome of 985 subjects was profiled. For details, see original work by Altman, Rinchai *et al*. ^16^ GEO deposition: https://www.ncbi.nlm.nih.gov/geo/query/acc.cgi?acc=GSE100150; plot: https://chart-studio.plotly.com/create/?fid=dchaussabel%3A132 interactive version with values expressed as counts: http://cd2k.gxbsidra.org/dm3/miniURL/view/NH.

We found differences in levels of CEACAM6 transcript abundance for nine of the 16 reference BloodGen3 datasets ( Table 4, **Extended Data File 6** ^57^). The pathological or physiological states for which differences were observed did not overlap with those also listed in Table 4 that were identified in the previous step by in depth screening of the literature. Indeed, we found elevated abundance levels of CEACAM6 in patients with infections caused by *Staphylococcus aureus*, influenza, respiratory syncytial virus, human immunodeficiency virus, and bacterial pathogens causing sepsis, in comparison with controls ( Figure 3). CEACAM6 transcript levels were not increased in patients with tuberculosis. Significant increases were also observed in non-communicable diseases such as systemic onset juvenile arthritis and Kawasaki disease but not in the context of systemic lupus erythematosus, late-stage melanoma, or chronic obstructive pulmonary disease. Finally, we also found a significant increase in abundance in the blood of liver transplant recipients under immunosuppressive therapy and in pregnant women. This transcriptome profiling dataset screen complemented our earlier literature screen, identifying nine additional diseases or physiological states in which CEACAM6 transcript is significantly changed in the blood of patients, for a total of 25 distinct diseases/states which are listed in Table 4. Plots for the nine BloodGen3 datasets are available via the GXB application and have been replotted and loaded to the CEACAM6 circle packing plot ( https://prezi.com/view/pQ7TKEC6tgY3cuik9ckt/ CEACAM6/Step5: blood tx profiling/CEACAM6_Blood Tx).

Overall, the screening of a reference dataset collection indicated that differences in CEACAM6 levels could be observed in a wide range of conditions in which systemic inflammation is observed. The lack of overlap between the literature and transcriptome data profiling conducted in steps 4 and 5 suggests that expanding this search to a larger number of blood transcriptome datasets would likely significantly add to this list.

### To date, no drugs have been developed that target CEACAM6

Another criterion for inclusion of CEACAM6 in a focused assay could be its targeting by approved drugs or drugs currently under development. The “Open Targets” database does not report any known drugs, approved or currently under development, targeting CEACAM6 ( https://platform.opentargets.org/target/ENSG00000086548). However, given its recently described role as suppressor of effector CD8 T-cells, ^28^ CEACAM6 is currently considered an immune checkpoint molecule and as such could be targeted by drugs designed to block its activity in cancer patients. ^27^ Additionally, in preclinical mouse models antibodies targeting CEACAM6 have been shown to inhibit tumor growth and metastasis. ^25^^,^^58^

### Profiling reference transcriptome datasets shows CEACAM6 transcript expression to be restricted to circulating neutrophils

Finally, screening of reference public transcriptome datasets can also yield insights regarding the candidate gene’s regulation and restriction among circulating leukocytes. Thus, in addition to profiling 16 public blood transcriptome datasets, we examined CEACAM6 transcriptional profiles in two other reference datasets. One dataset measured transcript abundance in monocytes, neutrophils, B-cells, CD4+ T-cells, CD8+ T-cells and natural killer (NK) cells and in whole blood (GSE60424 ^59^). The second dataset measured changes in transcript abundance in whole blood exposed *in vitro* to a wide range of immune stimuli (toll-like receptor agonists, killed bacteria, viruses, inflammatory cytokines and interferons; GSE30101 ^60^). In addition, we screened the Broad Institute’s single cell portal ^61^ for datasets in which CEACAM6 expression was elevated in one or more of the cell clusters.

**Figure 4.**
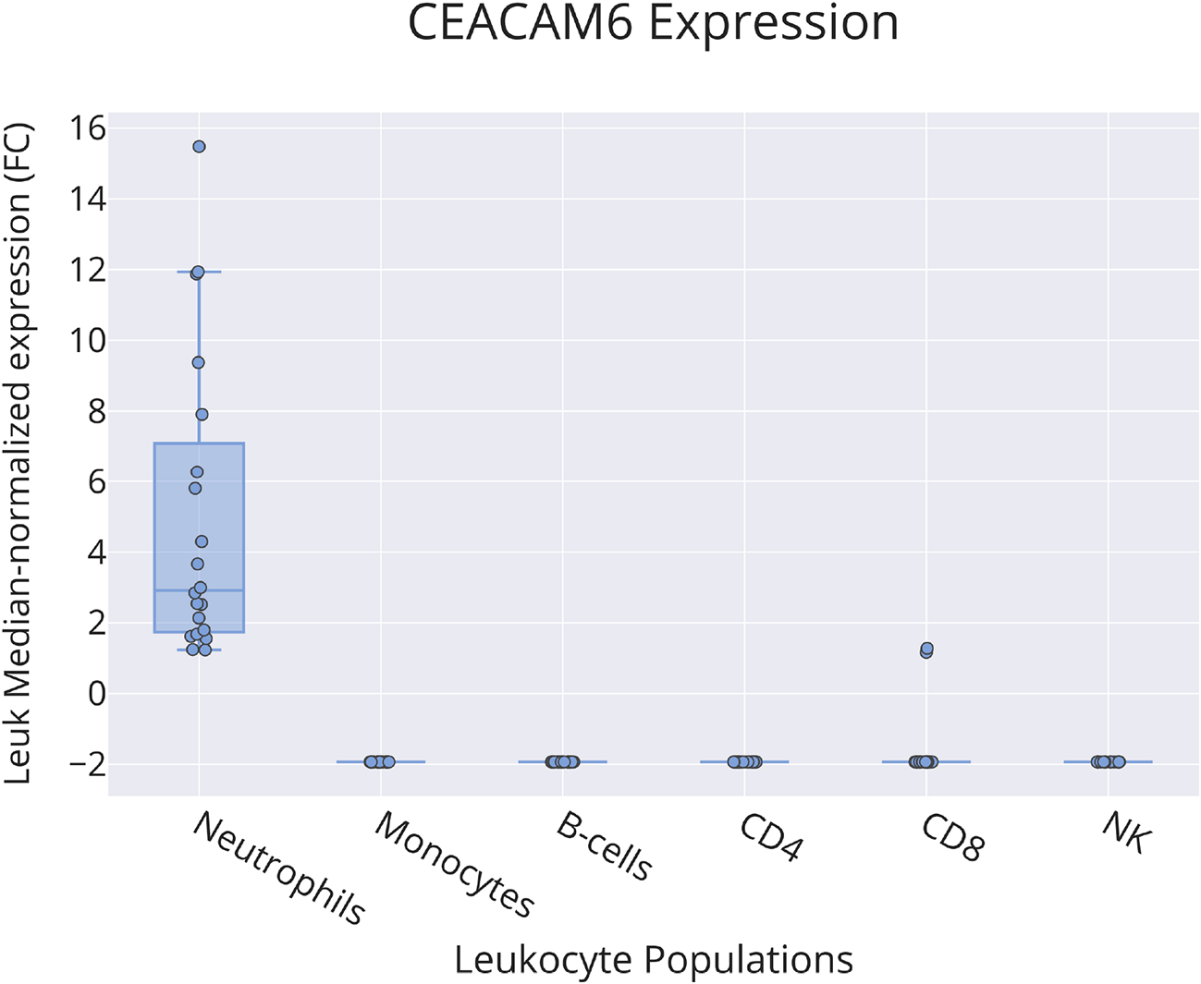
CEACAM6 restriction among circulating leukocyte populations. This box plot shows levels of abundance of CEACAM6 RNA measured by RNA sequencing in neutrophils, monocytes, B-cells, CD4+ T-cells, CD8+ T-cells and NK cells purified from the blood of human subjects, including patients with ALS, type 1 diabetes, multiple sclerosis (immediately before and 24 hours after initiation of beta interferon therapy) or sepsis and healthy controls. Values are normalized to the median calculated across all conditions. For details, see original work by Linsley *et al*. ^59^ GEO deposition: https://www.ncbi.nlm.nih.gov/geo/query/acc.cgi?acc=GSE60424 plot: https://plotly.com/~dchaussabel/171/.

**Figure 5.**
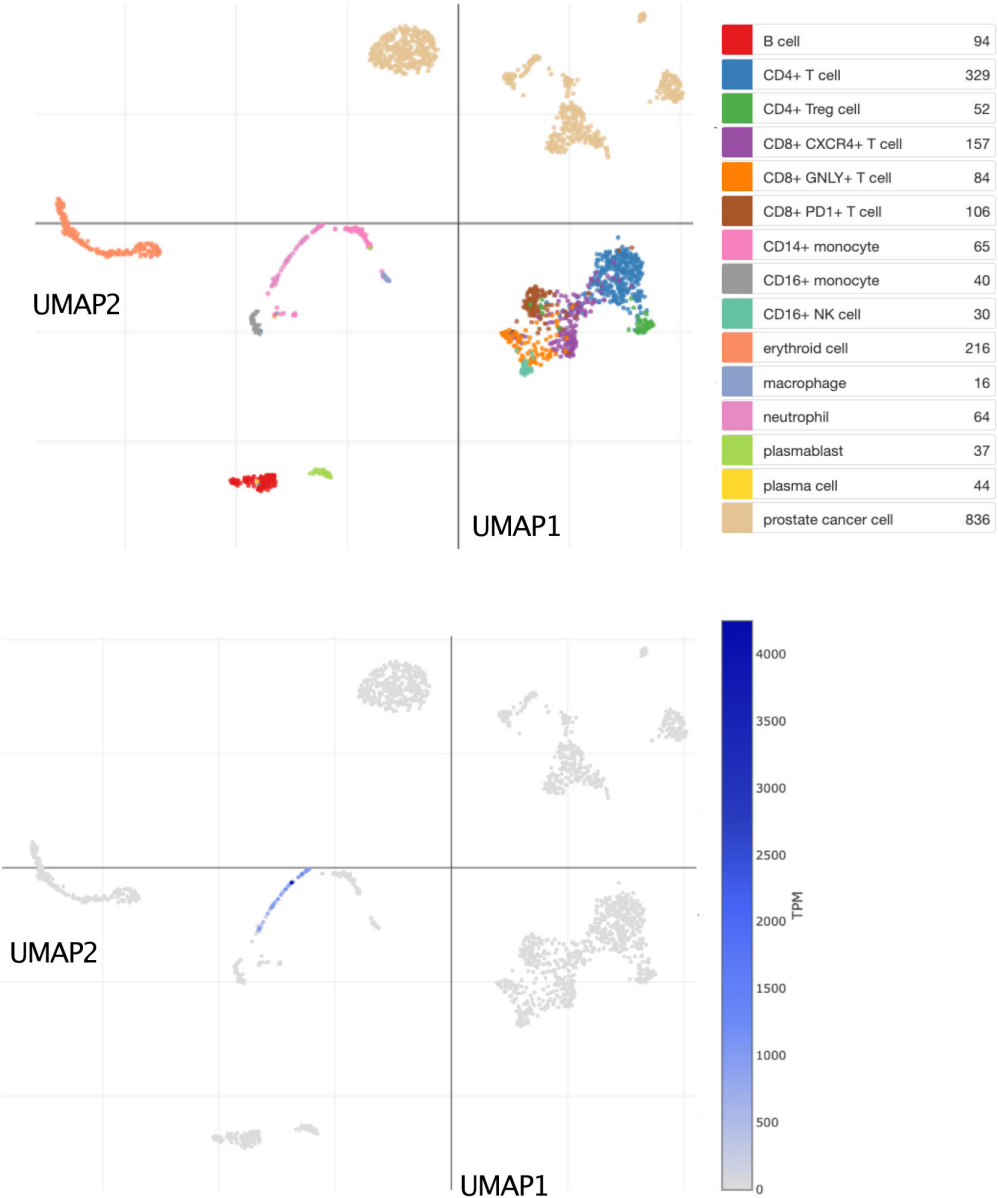
CEACAM6 expression at the single-cell level among dissociated prostate tumor tissue cells. This tSNE plot shows abundance levels of CEACAM6 measured by single-cell RNA sequencing among dissociated metastatic prostate tumor tissue cells. After quality control, this set consisted of 2,170 cells obtained from 14 patients and 15 biopsies. Clusters are labelled for dominant cell type based on marker gene expression on the plot above. Normalized transcript per million (TPM) counts for CEACAM6 are shown in blue on the plot below. For details, see original work by He *et al*. ^62^ An interactive version of this plot is accessible via the Broad Institute single cell portal: https://singlecell.broadinstitute.org/single_cell/study/SCP1244/transcriptional-mediators-of-treatment-resistance-in-lethal-prostate-cancer?genes=CEACAM6#study-visualize.

**Figure 6.**
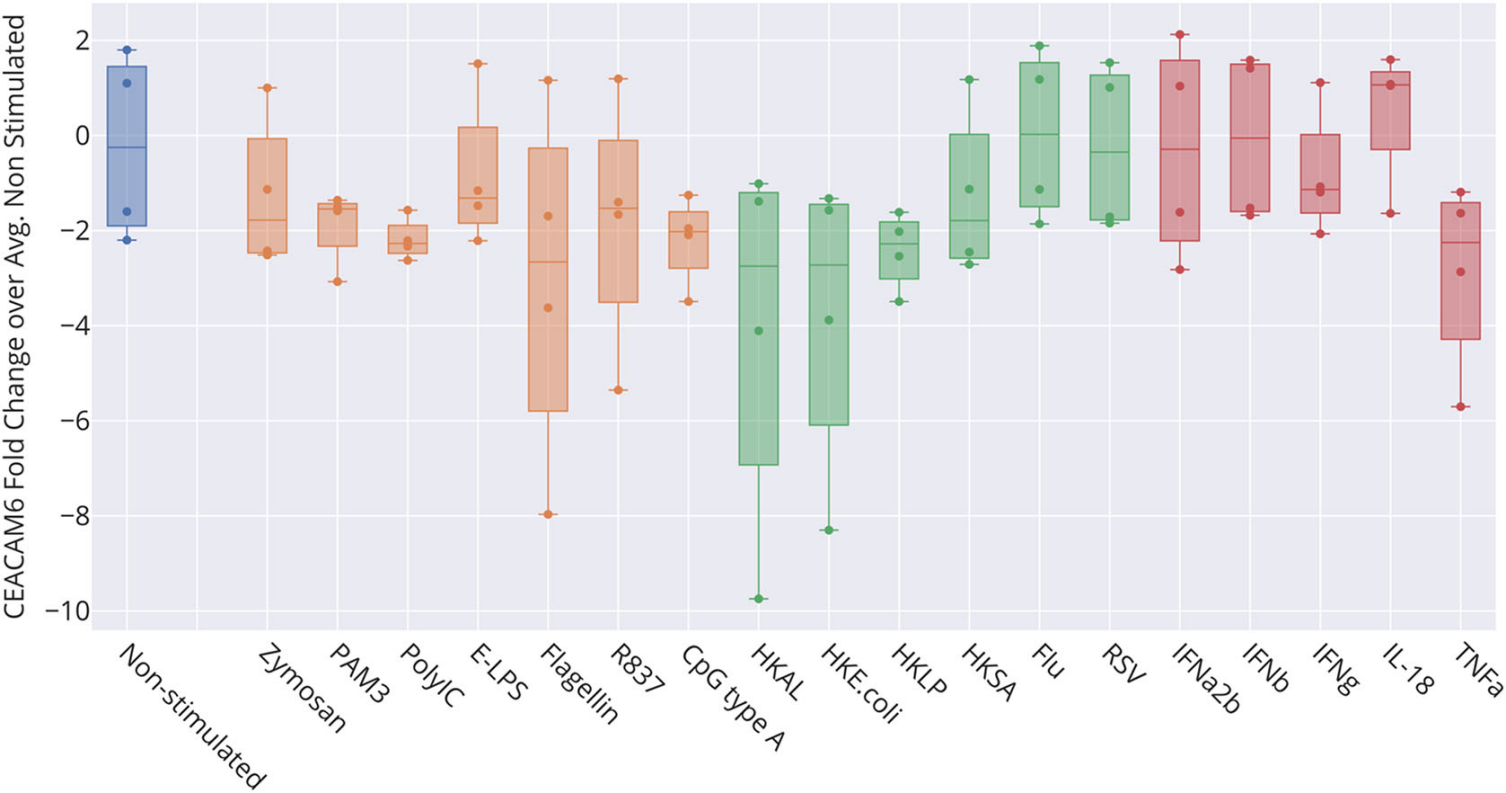
Levels of CEACAM6 transcript abundance in blood exposed to immune stimuli *in vitro.* The box plot represents transcript abundance levels for the CEACAM6 gene in blood samples obtained from four healthy donors, cultivated for 6 hours at 37°C in the presence of pathogens, as well as pathogen-derived and host-derived immune stimuli (HKAL=heat-killed *Acholeplasma laidlawii*, HKLP=heat-killed *Legionella pneumophila*, HKSA=heat-killed *Staphylococcus aureus*, Flu=live influenza A virus, RSV=live respiratory syncytial virus). For details, see original work by Obermoser *et al*. ^60^ GEO deposition: https://www.ncbi.nlm.nih.gov/geo/query/acc.cgi?acc=GSE30101 plot: https://plotly.com/~dchaussabel/173/.

Bulk leukocyte population RNAseq data showed CEACAM6 expression to be restricted to neutrophils ( Figure 4) [data source: Linsley *et al*. ^59^]. This observation was confirmed in a single-cell dataset in which tumor immune cell infiltrates were dissociated and profiled via RNA sequencing ( Figure 5) [data source: He *et al*. ^62^]. These findings were in line with the prevalence among the CEACAM6 literature of publications mentioning this cell type ( https://prezi.com/view/pQ7TKEC6tgY3cuik9ckt/ CEACAM6/Step 3: background literature profiling/CEACAM6_Cell Types) ( Figure 2A). However, we did not find CEACAM6 to be increased in whole blood stimulated *in vitro* ( Figure 6) [data source: Obermoser *et al*. ^60^]. This finding was to some extent surprising since blood signatures comprising CEACAM6 are often functionally associated with neutrophil activation. ^63^^–^^65^

Taken together, further profiling of reference transcriptome datasets confirmed the close association of CEACAM6 with neutrophils, which is the most abundant circulating leukocyte population in blood. It also indicates that elevated levels of CEACAM6 transcript abundance observed across a wide range of conditions may be associated with an increase in relative abundance of cells expressing this gene, rather than regulation of its expression.

## Discussion

Clinical translation of biomarker signatures obtained via transcriptome profiling technologies typically involves the development of targeted transcript panels and assays. Such assays can also prove more practical for high-temporal frequency immunological monitoring applications that require profiling of thousands of samples. They could also be more readily implemented in the context of research projects conducted in low-resource settings. Targeted panel design can be informed by both data-driven and knowledge-driven approaches. However, given the large amounts of data and knowledge available for any given candidate gene, the selection process can prove daunting. Here we employed a workflow devised for screening the literature and large-scale profiling data associated with a given candidate gene, and to retrieve and aggregate relevant information in a structured format. This information and associated resources should in turn support decision-making of investigators aiming to develop targeted panels for downstream clinical or research applications.

We focused on CEA cell adhesion molecule 6 (CEACAM6). This candidate is a member of blood transcriptional signatures that are often functionally associated with neutrophil activation, ^63^^–^^65^ which typically also includes genes encoding constituents of neutrophil granules, such as defensins (DEFA1, DEFA3, DEFA4), myeloperoxidase (MPO), bactericidal permeability increasing protein (BPI), and lactotransferrin (LTF).

Several criteria can be used when prioritizing candidate genes for inclusion in a targeted assay, which we have applied here to CEACAM6:

1) Transcripts are detectable in blood and changes can be observed across different immune states/pathologies; this criterion is met in the case of CEACAM6. An increase in levels of CEACAM6 transcripts has been reported in the literature and observed in blood transcriptome datasets for patients with infectious (e.g., bacterial sepsis), autoimmune, or inflammatory diseases (e.g., systemic lupus erythematosus, Kawasaki disease).
2) Previous reports describe the candidate as being of clinical relevance as a biomarker; this criterion is also met. Indeed, CEACAM6 is part of a family that includes members which are used routinely in clinical pathology to assess tumor specimens and inform disease prognosis and treatment. ^66^^–^^68^ CEACAM6 itself is deemed of potential value as a prognosis marker in different types of cancers. ^32^^,^^33^^,^^37^^,^^39^^,^^40^ Notably, measuring blood CEACAM6 transcript abundance is considered of potential value for the early detection of colorectal cancer. ^14^^,^^15^
3) The functional relevance of the candidate gene in blood leukocytes is known; this criterion is partially met. CEACAM6 is associated with neutrophils in the literature. This was confirmed in our screen of reference transcriptome datasets, both at the bulk leukocyte population and single cell levels ( Figures 4 & 5). However, the role played by CEACAM6 in neutrophils has not yet been fully elucidated. For instance, another reference dataset showed that CEACAM6 expression is not regulated in blood exposed *in vitro* to a wide range of immune stimuli ( Figure 6). This finding casts some doubts on whether “neutrophil activation” should be assigned to the signature associated with CEACAM6 (by us and others). These observations may also be consistent with an earlier report that associated a “granulopoiesis signature”, which comprised CEACAM6, with low density mononuclear and polymorphonuclear populations found in peripheral blood mononuclear cell fractions. ^69^ Furthermore, single-cell analyses recently conducted in COVID-19 patients identified a population of “developing neutrophils” that expressed neutrophil granule proteins, including module M10.4 members such as MPO, DEFA3, LTF, and ELANE, and were described as potentially being derived from plasmablasts. ^70^ Altogether these observations suggest that measuring levels of M10.4 transcripts might permit the monitoring of changes in abundance in this population of developing neutrophils rather than reflecting overall neutrophil abundance. However, this hypothesis and the functional relevance of this subset of neutrophils remains to be validated experimentally.
4) The candidate gene is a target for drugs that are approved or under development; this criterion is not met. No drugs targeting CEACAM6 have been developed to date. This may change, however since, as noted earlier, CEACAM6 has recently been found to suppress T-cell function and could thus be considered for targeting by novel immune checkpoint inhibitors in cancer patients. ^28^

Alternate candidates may be found that could be selected instead of CEACAM6 for inclusion in a targeted blood transcriptional assay. CEACAM6 was chosen for this evaluation based on its membership to module M10.4, which is part of the fixed BloodGen3 repertoire. ^17^ Such module repertoires can be employed as a framework for the design of targeted assays, in which case only one or a few representative transcripts from a given module would usually be selected to provide coverage for the entire repertoire (those modules are formed based on co-expression and all constitutive transcripts would present with a high degree of co-linearity). ^9^ In the case of module M10.4, other candidates to consider would be CEACAM8, BPI, MPO, LTF, DEFA1, DEFA3, DEFA4, CTSG, OLFM4, and ELANE, since all of those genes belong to the same module as CEACAM6 ( Table 5). However, to date, only CEACAM6 has been investigated in depth and thus it is not yet possible to benchmark it against these other candidates. However, it can already be noted that BPI (bactericidal/permeability-increasing protein) has been found to be of potential value as a biomarker in patients with asthma, ^71^ as well as chronic obstructive pulmonary disease. ^72^ DEFA1 and DEFA3 have been identified as potential inflammatory biomarkers for coronary heart disease. ^73^ CEACAM8, another member of the carcinoembryonic cell adhesion molecule family, has been found to be of potential value as a prognosis marker in patients with esophageal cancer and in patients with sepsis. ^74^^,^^75^

**Table 5.** Published gene signatures comprising CEACAM6. This table lists targeted gene sets or gene panels comprising CEACAM6. Lists of differentially expressed genes that consists of tens or hundreds of transcripts are purposedly omitted.

Disease/physiological state	Signature name or description	Gene set	PMID/Reference
Multiple	BloodGen3/M10.4	MPO, LTF, BPI, CEACAM6, CEACAM8, DEFA1, DEFA3, DEFA4, CTSG, ELANE, OLFM4	34282143 ^16^
Parkinson’s disease		ADARB2, CEACAM6, CNTNAP2, COL19A1, DEF4, DRAXIN, FCER2, HBG1, NCAPG2, PVRL2, SLC2A14, SNCA, and TCL1B	25475535 ^44^
Colorectal cancer	CELTiC Panel	LGALS4, CEACAM6, TSPAN8, COL1A2	29352642 ^14^
Idiopathic pulmonary fibrosis		CAMP, CEACAM6, CTSG, DEFA3 DEFA4, OLFM4, HLTF, PACSIN1, GABBR1, IGHM	22761659 ^45^
Lung cancer		HK3, SLC36A1, MSR1, CEACAM1, CEACAM6, HCG27, FXYD7, TRPLC1, NR3C2, RLN2	34288383 ^43^
COVID-19	Neutrophil-associated gene cluster	CEACAM6, RETN, MPO, LTF, MMP8, CEACAM8, DEFA4, OLR1, DEFA3, DEFA1B, DEFA1, ELANE	34335605 ^51^
COVID-19	Secretory granules signature	CEACAM8, MMP8, ELANE, LTF, CEACAM6, MPO	35844004 ^52^

Finally, it is worth highlighting some of the limitations of our investigation into the relevance of CEACAM6 as a blood transcriptome biomarker. For instance, it should be noted that the screen conducted among public transcriptome data is not comprehensive. Additional blood transcriptome datasets are available in GEO and other repositories that have not yet been loaded in GXB instances. As a result, the list of conditions in which CEACAM6 blood transcript abundance changes is probably conservative and will likely grow as more datasets become available for screening.

In conclusion, the information presented here should help researchers decide whether to include CEACAM6 in the targeted assay they intend to develop. Some of our findings suggest that measuring abundance of CEACAM6 transcripts in blood could prove to be of value in the monitoring and management of patients with diseases associated with systemic inflammation. This would likely be true for other members of the BloodGen3 module M10.4/“neutrophil activation” gene sets. However, CEACAM6 presents with the distinct advantage of also being of potential value in the management of patients with cancer, whether the assay would be used to measure transcript abundance in blood or in tumor tissues.

## Author contributions

DR and DC: Conceptualization, Data curation, Formal analysis, Visualization, Methodology Development, Writing – Review & Editing. DC: Writing – Original Draft Preparation. The contributor's roles listed above follow the Contributor Roles Taxonomy (CRediT) managed by The Consortia Advancing Standards in Research Administration Information (CASRAI) ( https://casrai.org/credit/).

## Data Availability

The project contains the following extended data:
•
**Extended Data File 1:** a spreadsheet in the MS Excel format that is used as a template to capture relevant information from the literature and from transcriptional profiling data analysis results. Figshare: Ext Data File 1 - Information Capture Form_Generic_2022 Sept14 https://doi.org/10.6084/m9.figshare.21183718.v1. ^19^
•
**Extended Data File 2**: a spreadsheet in the MS Excel format listing cell type and disease entities and their prevalence in the literature associated with CEACAM6. Figshare: Ext Data File 2 CEACAM6_Lit Profiles_Entities_Step3c_2022 Sept14 https://doi.org/10.6084/m9.figshare.21183748.v1. ^29^
•
**Extended Data File 3**: a spreadsheet in the MS Excel format used to capture information from the CEACAM6 literature regarding its actual or potential use as a biomarker. Figshare: Ext Data File 3 CEACAM6_Articles_Biomarker Relevance_Step3d_2022 Sept14. https://doi.org/10.6084/m9.figshare.21183832.v1. ^30^
•
**Extended Data File 4**: a spreadsheet in the MS Excel format used to capture information from the CEACAM6 literature reporting differences in blood transcript abundance in cases vs controls. Figshare: Ext Data File 4 CEACAM6_Articles_Blood transcript profiling_Step4c_2022 Sep14. https://doi.org/10.6084/m9.figshare.21184357.v1. ^55^
•
**Extended Data File 5**: a spreadsheet in the MS Excel format used to capture CEACAM6 transcriptional profiles from multiple datasets (one dataset per tab) and compute significance of differences in abundance observed between cases and controls. Figshare: Ext Data File 5 CEACAM6_Transcriptome data_ abundance profiles_Step5b_2022 Sept14. https://doi.org/10.6084/m9.figshare.21184363.v1. ^56^
•
**Extended Data File 6**: a spreadsheet in the MS Excel format used to capture relevant information regarding differences in CEACAM6 blood transcriptional abundance observed in multiple datasets. Figshare: Ext Data File 6 CEACAM6_Transcriptome data_diff expression_Step5c_2022 Sept14. https://doi.org/10.6084/m9.figshare.21184369.v1. ^57^ Data are available under the terms of the Creative Commons Attribution 4.0 International license (CC-BY 4.0).
