# Supplementary material for: Assessing the potential relevance of CEACAM6 as a blood transcriptional biomarker
Source: F1000Res. 2024 Apr 4;11:1294. Originally published 2022 Nov 11. [Version 2] doi: 10.12688/f1000research.126721.2 (PMC11375406; doi:10.12688/f1000research.126721.2)
Supplement: Peer review history

# Reviewer report: Reviewer response for version 2

**DOI:** 10.5256/f1000research.163578.r289474

**Published:** 2024-09-04

**Authors:** Akila Prashant, Deepthi V

**Affiliations:** 1 Biochemistry, JSS Academy of Higher Education & Research, Mysore, Karnataka, India; 2 Biochemistry, JSS Academy of Higher Education and Research, Mysuru, Karnataka, India

This article offers comprehensive and detailed information on the process of screening a protein as a potential biomarker. It is assumed that this method can be applied to any protein for screening, assessment, and correlation with various diseases. 

 While the paper provides methods for screening a biomarker, it lacks clarity regarding its purpose. It is unclear whether the objective is to explain the screening methods, or to demonstrate CEACAM6 as a potential biomarker, or both.

 If it is both:

1. The author is suggested to represent the flowchart of the methodology section in detail.
2.  The paper lacks an explanation of  CEACAM6 as a protein and its physiological functions/ pathways in the context of diseases since the title claims to reveal the potential relevance of CEACAM6.
3. The paper is expected to balance both the methodology of biomarker screening as well as CEACAM6 functions and potentiality as a protein as well as biomarker.

Is the work clearly and accurately presented and does it cite the current literature?

Yes

If applicable, is the statistical analysis and its interpretation appropriate?

Yes

Are all the source data underlying the results available to ensure full reproducibility?

Yes

Is the study design appropriate and is the work technically sound?

Yes

Are the conclusions drawn adequately supported by the results?

Yes

Are sufficient details of methods and analysis provided to allow replication by others?

Yes

Reviewer Expertise:

Rare Disease Genetics, Cancer Biology

We confirm that we have read this submission and believe that we have an appropriate level of expertise to confirm that it is of an acceptable scientific standard, however we have significant reservations, as outlined above.

# Reviewer report: Reviewer response for version 2

**DOI:** 10.5256/f1000research.163578.r263048

**Published:** 2024-04-13

**Authors:** Ritu Pandey, Daruka Mahadevan

**Affiliations:** 1 Department of Cellular and Molecular Medicine, University of Arizona, Tucson, USA; 2 The University of Texas Health Science Center (Ringgold ID: 12346), San Antonio, Texas, USA

No further comments.

Is the work clearly and accurately presented and does it cite the current literature?

Partly

If applicable, is the statistical analysis and its interpretation appropriate?

Yes

Are all the source data underlying the results available to ensure full reproducibility?

Partly

Is the study design appropriate and is the work technically sound?

Yes

Are the conclusions drawn adequately supported by the results?

Yes

Are sufficient details of methods and analysis provided to allow replication by others?

Partly

Reviewer Expertise:

Cancer research

We confirm that we have read this submission and believe that we have an appropriate level of expertise to confirm that it is of an acceptable scientific standard.

# Reviewer report: Reviewer response for version 1

**DOI:** 10.5256/f1000research.139158.r192786

**Published:** 2023-10-03

**Authors:** Ritu Pandey, Daruka Mahadevan

**Affiliations:** 1 Department of Cellular and Molecular Medicine, University of Arizona, Tucson, USA; 2 The University of Texas Health Science Center (Ringgold ID: 12346), San Antonio, Texas, USA

The manuscript is a summary of CEACAM6 gene as a prognostic blood biomarker across diseases and provides a guide to capture heterogeneous information on a given gene or biomarker using publicly available processed or summarized data. The work is based on prior published work by the group on analysis of blood biomarkers.

  It is not clear that the main focus of the paper is showing a method or in-depth analysis of CEACAM6 gene utilizing the method, assuming both here are few comments:

1. The authors provide for the readers a pathway to gather details on a gene and summarize the information. But it is not clear how the information is getting extracted. The data captured is voluminous at times, so are there any scripts that the authors are providing or suggesting for data retrieval? There is some amount of customized data massaging and data wrangling needed for further use, but few automated steps and scripts would be useful. Data backed with evidence keeps changing such as PubMed and needs to be updated too. The strategy is based on previous work by the authors but any automation or programmatic retrieval for any of the steps should be included here for the benefit of readers.
2. The statement in the Discussion #4 that no drugs have been developed for targeting CEACAM6 could be misleading since there have been efforts, at least in cancer studies to target CEACAM6 using monoclonal antibodies. The authors do mention one myeloma study. There are several experimental approaches that have been published in preclinical models and there are also clinical studies in cancer where this is being targeted as an immune checkpoint. 
3. This study assesses changes in expression of any gene in disease cases to qualify it as a marker but equally important is what approaches have been used or what indications exist that it has been studied as a therapeutic target and if so the outcome of such study. There are several publications for studies of CEACAM6 as a therapeutic target in cancer. Examples of few of the published work from NCBI PubMed - PMID: 19334050, PMID: 35141051, PMID: 31797958, PMID: 35082925 and a clinical trial - https://clinicaltrials.gov/study/NCT03596372. These should be cited as similar approaches could be utilized for other diseases.

Is the work clearly and accurately presented and does it cite the current literature?

Partly

If applicable, is the statistical analysis and its interpretation appropriate?

Yes

Are all the source data underlying the results available to ensure full reproducibility?

Partly

Is the study design appropriate and is the work technically sound?

Yes

Are the conclusions drawn adequately supported by the results?

Yes

Are sufficient details of methods and analysis provided to allow replication by others?

Partly

Reviewer Expertise:

Cancer research

We confirm that we have read this submission and believe that we have an appropriate level of expertise to confirm that it is of an acceptable scientific standard, however we have significant reservations, as outlined above.

## References

[1] : Preclinical evaluation of carcinoembryonic cell adhesion molecule (CEACAM) 6 as potential therapy target for pancreatic adenocarcinoma. *J Pathol* .2009;218(3) : 10.1002/path.2545 380-90 10.1002/path.2545 19334050

[2] : T cell-mediated elimination of cancer cells by blocking CEACAM6-CEACAM1 interaction. *Oncoimmunology* .2022;11(1) : 10.1080/2162402X.2021.2008110 2008110 10.1080/2162402X.2021.2008110 35141051 PMC8820806

[3] : Carcinoembryonic antigen cell adhesion molecule 6 (CEACAM6) in Pancreatic Ductal Adenocarcinoma (PDA): An integrative analysis of a novel therapeutic target. *Sci Rep* .2019;9(1) : 10.1038/s41598-019-54545-9 18347 10.1038/s41598-019-54545-9 31797958 PMC6893022

[4] : Prognostic value of the immune target CEACAM6 in cancer: a meta-analysis. *Ther Adv Med Oncol* .2022;14: 10.1177/17588359211072621 17588359211072621 10.1177/17588359211072621 35082925 PMC8785271

# Response

**Published:** 2024-03-08

**Authors:** Damien Chaussabel

**Affiliations:** The Jackson Laboratory for Genomic Medicine, USA

Thank you for your constructive review and insightful comments. We understand the concerns raised regarding the clarity of our manuscript's focus and the methodologies employed for data retrieval and analysis. Allow us to address these points with additional context that we believe will clarify our intentions and the contributions of our work.

**Clarification of Manuscript Focus**: Our current work is intended as a proof of concept paper, demonstrating the practical application of the methodological framework we detailed in a previously published paper ("A training curriculum for retrieving, structuring, and aggregating information derived from the biomedical literature and large-scale data repositories"). This earlier publication provides an in-depth description of the methods and serves as the foundation upon which our current study on CEACAM6 is built. We acknowledge that this may not have been made sufficiently clear in our manuscript, leading to confusion about its primary focus. We revised our introduction sections to explicitly state that this work is a demonstration of our previously published methodology applied to the CEACAM6 gene, rather than an exposition of new methodological advancements:

*“The methodology employed in this study is derived from our previously established “collective omics data” (COD) training curriculum, as outlined in our comprehensive methods paper, "A training curriculum for retrieving, structuring, and aggregating information derived from the biomedical literature and large-scale data repositories." ^10^. This foundational paper provides a detailed description of our systematic approach to information curation, which we have applied in the current investigation of CEACAM6. Specifically, the study utilizes the COD1 training module workflow from this curriculum, which guides the structured retrieval and aggregation of gene-specific data for biomarker assessment. The process encompasses selecting a gene of interest, in this case, CEACAM6, to comprehensively gather and synthesize relevant information from both literature and public datasets, culminating in the creation of resources like structured data tables and interactive circle packing plots. This approach not only supports the rigorous assessment of CEACAM6's potential as a blood biomarker but also serves as a demonstrative application of our validated methodological framework, providing a practical example of how such a framework can be employed to enhance biomarker discovery efforts.”*

**Data Retrieval and Analysis**: The method described in our prior paper involves a systematic but manual approach to retrieving, structuring, and aggregating information, which, as you rightly pointed out, can be labor-intensive. We recognize the importance of automation in managing the vast amount of data available in biomedical research. While our published method does not incorporate automated processes, we have, in response to the evolving needs of data curation, begun exploring the potential of Large Language Models (LLMs) to assist in manual data curation tasks. This effort is led by staff who have recently joined our group and represents an exciting direction for enhancing the efficiency and scalability of our data curation processes. Preliminary findings suggest that while LLMs are not a complete substitute for manual curation, they can significantly aid in the process by streamlining the identification and extraction of relevant information. This ongoing work acknowledges the pertinence of your feedback regarding automation and highlights our commitment to advancing our methodologies in line with technological developments. Although the results of these explorations are not included in the current manuscript, they are part of a separate study that we plan to publish in the future. This will detail our experiences and findings regarding the integration of LLMs into our data curation workflow, providing insights that could benefit the broader research community in handling similar challenges.A new paragraph has been added to the discussion, acknowledging current limitations and potential strategies for automating the information extraction workflow:

*“The current methodology reliance on a systematic, manual approach to data retrieval and structuring is another limitation. We recognize the potential of automation to transform this labor-intensive process. In this respect, we are actively exploring the integration of Large Language Models (LLMs) into our data curation workflow. These advanced models show promise in streamlining the identification, extraction, and structuring of relevant information, potentially mitigating the challenges associated with the sheer volume and dynamic nature of biomedical databases. Our preliminary explorations suggest that while LLMs may not fully replace the nuanced judgment of human curators, they offer significant support by enhancing efficiency and accuracy, thereby complementing our existing methodologies. Thus we are cautiously optimistic about the role of LLMs in enhancing our data analysis framework, aiming to improve efficiency while maintaining accuracy. This integration of LLMs is an ongoing effort and will be detailed further in upcoming publications.”*

**Discussion on Therapeutic Targeting of CEACAM6**: We appreciate your pointing out the need to correct and expand our discussion on therapeutic efforts targeting CEACAM6. It was not our intention to overlook significant research in this area. We have revised the relevant sections to accurately reflect ongoing and completed studies targeting CEACAM6 with therapeutic intent, citing the publications and clinical trials you mentioned. This will ensure our discussion acknowledges both the biomarker potential of CEACAM6 and its implications for therapeutic development:

*Recent studies and ongoing clinical trials have explored the utility of targeting CEACAM6 in various cancers, particularly through the development of monoclonal antibodies. For instance, preclinical evaluations have demonstrated the potential of CEACAM6 as a therapy target in pancreatic adenocarcinoma, utilizing antibody-drug conjugates to effectively target and diminish CEACAM6-expressing tumors (PMID: 19334050). Additionally, the blocking of CEACAM6-CEACAM1 interactions has shown promise in enhancing T cell-mediated cancer cell elimination, suggesting a role for CEACAM6 in immune modulation and its potential as an immune checkpoint target (PMID: 35141051). The breadth of research, encompassing studies on its prognostic value and therapeutic targeting in cancers, underscores CEACAM6's significance in oncology and its emerging role as a viable therapeutic target. These investigations, reflected in various studies (PMID: 31797958, PMID: 35082925) and a clinical trial registered under NCT03596372, collectively indicate a growing interest in CEACAM6 as a therapeutic target, warranting further exploration and validation in clinical settings.*

 In summary, we are committed to improving the clarity and utility of our manuscript based on your feedback. We believe that by clarifying the relationship between our current work and our previously published methodological framework, and by addressing the points you've raised regarding data analysis and therapeutic targeting, our manuscript will provide a clearer, more comprehensive contribution to the field. Thank you again for your valuable insights, which we are confident will strengthen our paper.

# Reviewer report: Reviewer response for version 1

**DOI:** 10.5256/f1000research.139158.r155597

**Published:** 2022-12-09

**Authors:** Mattia Lauriola

**Affiliations:** 1 Department of Experimental, Diagnostic and Specialty Medicine, University of Bologna, Bologna, Italy

This article is quite interesting, because the authors highlight a possible role for CEACAM6 as a blood biomarker in patients with a viral or bacterial infection, thus surrogating CEAM6 as an assay for systemic inflammation. As the authors correctly pointed out, CEACAM6 transcript levels were already described for cancer diseases including colorectal cancer. In this case, it's interesting to notice that previous publications reported that CEACM6 abundance in the blood of subjects positive for FIT (faecal immunochemical test), but negative for further intestinal lesions. This probably finds support in some of the results reported here, showing an enhanced availability of CEACAM6 in circulating neutrophils. But, the authors fail to refer to this publication PMID: 32257432 ^1^.

Is the work clearly and accurately presented and does it cite the current literature?

Partly

If applicable, is the statistical analysis and its interpretation appropriate?

I cannot comment. A qualified statistician is required.

Are all the source data underlying the results available to ensure full reproducibility?

Yes

Is the study design appropriate and is the work technically sound?

Yes

Are the conclusions drawn adequately supported by the results?

Yes

Are sufficient details of methods and analysis provided to allow replication by others?

Yes

Reviewer Expertise:

Blood biomarkers for colon cancer, EGFR, RTK signalling

I confirm that I have read this submission and believe that I have an appropriate level of expertise to confirm that it is of an acceptable scientific standard, however I have significant reservations, as outlined above.

## References

[1] : Colorectal cancer screening: Assessment of CEACAM6, LGALS4, TSPAN8 and COL1A2 as blood markers in faecal immunochemical test negative subjects. *J Adv Res* .2020;24: 10.1016/j.jare.2020.03.001 99-107 10.1016/j.jare.2020.03.001 32257432 PMC7113625

# Response

**Published:** 2024-03-08

**Authors:** Damien Chaussabel

**Affiliations:** The Jackson Laboratory for Genomic Medicine, USA

Thank you for highlighting the importance of referencing the study associated with PMID: 32257432. We agree that this publication is relevant to our research on CEACAM6 as a potential blood biomarker. In light of your suggestion, we have added a reference to this study in our manuscript (now cited as reference #16). This inclusion helps to contextualize the role of CEACAM6 in subjects with a positive fecal immunochemical test (FIT) but no intestinal lesions, and supports our findings on its availability in circulating neutrophils. We appreciate your constructive feedback and believe that this addition enhances the clarity and relevance of our work.
